# Repeated Stereotactic Radiosurgery in Brain Metastases: A Case Report

**DOI:** 10.7759/cureus.2005

**Published:** 2017-12-30

**Authors:** Elena Configliacco, Liliana Belgioia, Salvina Barra, Flavio Giannelli, Stefano Agostinelli, Renzo Corvò

**Affiliations:** 1 Department of Radiation Oncology, IRCCS Policlinico San Martino, Genoa; 2 Radiation Oncology, University of Genoa Ospedale Policlinico San Martino; 3 Department of Radiation Oncology, IRCCS Ospedale San Martino, Genoa; 4 Medical Physics, IRCCS San Martino-IST, University-Hospital; 5 Department of Radiation Oncology, University of Genoa Ospedale Policlinico San Martino

**Keywords:** stereotactic radiosurgery, helical tomotherapy, nsclc

## Abstract

The aims of radiation therapy of brain metastases include maintaining neurocognitive function and control of disease and, hopefully, improvement of survival. We present a case report with a very long survival in which the role of repeated stereotactic radiosurgery (SRS) was investigated for a patient with a recurrent brain metastasis from non-small cell lung cancer in the same area. Stereotactic re-irradiation was successful and well-tolerated with no neurological toxicity after 16 months.

## Introduction

Non-small cell lung cancer (NSCLC) is the main source of brain metastases; about 30-50% of patients with NSCLC develop a brain metastasis during the course of their disease. Radiation therapy is one of the treatment options for these patients, however, treatment results for brain metastases from NSCLC are often not satisfactory considering the oncological outcome and the neurocognitive toxicity [1]. Here we present a case report of a patient with recurrent brain metastasis from NSCLC who had long survival after treatment with repeated stereotactic radiosurgery (SRS).

## Case presentation

**Figure 1:**
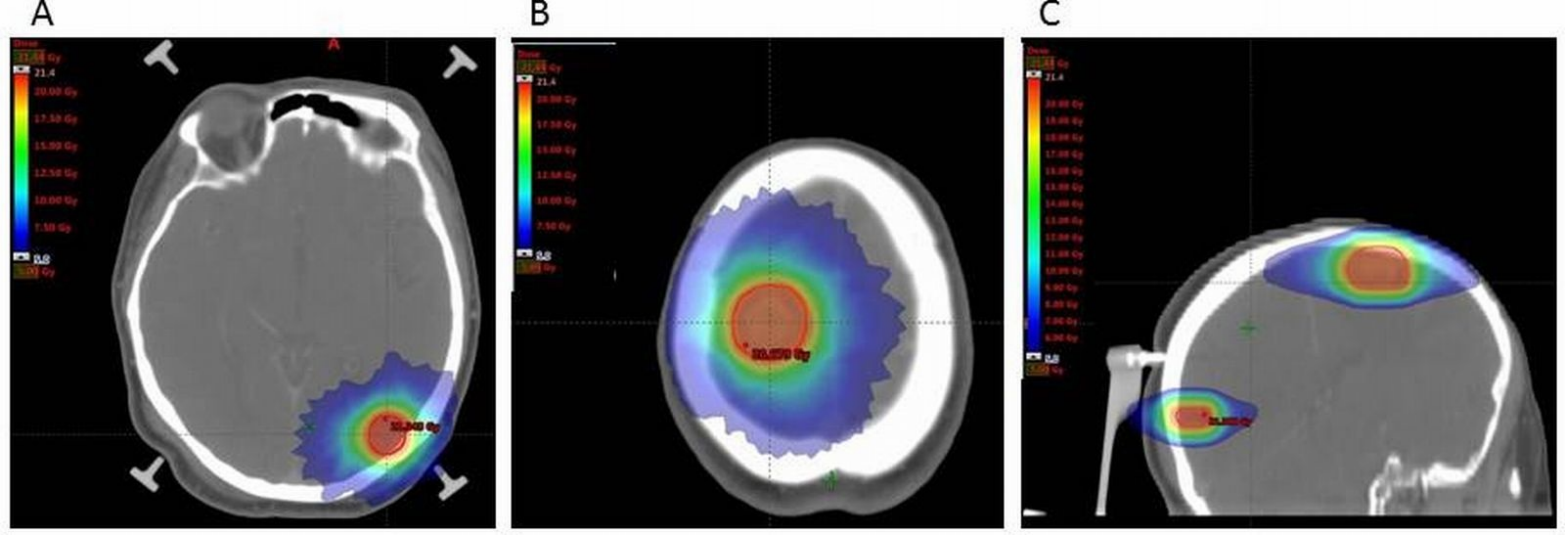
Dose distribution. Relative radiation dose levels are indicated by the colourwash (dose range: 21-5 Gy). Axial section for 1A: left occipital lesion, 1B: right frontal lesion. 1C: sagittal section for both lesions. Red line indicates PTV. PTV: Planning target volume.

In September 2011 a 52-year-old woman accessed our emergency department due to a progressive decreased level of consciousness, convulsion and sphincter’s release. Her pathological history reported an NSCLC treated seven months previously with neoadjuvant cisplatin (CDDP) based chemotherapy and upper left lobectomy. A computed tomography (CT) scan revealed one heterogeneous lesion measuring 1 cm in diameter at the right frontal lobe. Brain magnetic resonance imaging (MRI) detected two solid lesions, the first at the right frontal lobe of 1.9 cm in diameter and the second at the left occipital of 1 cm with surrounding edema and mass effect suggestive of metastasis. The woman received immediately dexamethasone 8 mg two times a day to relieve the symptoms and in October 2011 accessed our radiation therapy department for evaluation. To complete her staging she underwent a total body CT that revealed no other disease location. Thereafter, she received a single-fraction SRS on the two brain lesions. SRS was delivered by Hi-Art helical tomotherapy (HT) and planned using the 1 cm field width on a CT scan with a 2.5 mm slice thickness from the vertex to the second cervical vertebra. Dose prescription was hard constrained so that planning target volume’s (PTV) median dose equals the prescription dose. The patient was immobilized using the InterFix Radiosurgery Kit which fixes the patient’s skull to tomotherapy treatment couch. CT images were fused with MRI with 1 mm slice thickness, performed the day before on a GE SIGNA 3T scanner, to allow a better identification of the target and organs at risk (OAR). The clinical tumor volume (CTV) was expanded with an isotropic margin of 3 mm to obtain the PTV. Prescription dose was 21 Gy in a single fraction for each lesion (Figure 1A-1C).

**Figure 2:**
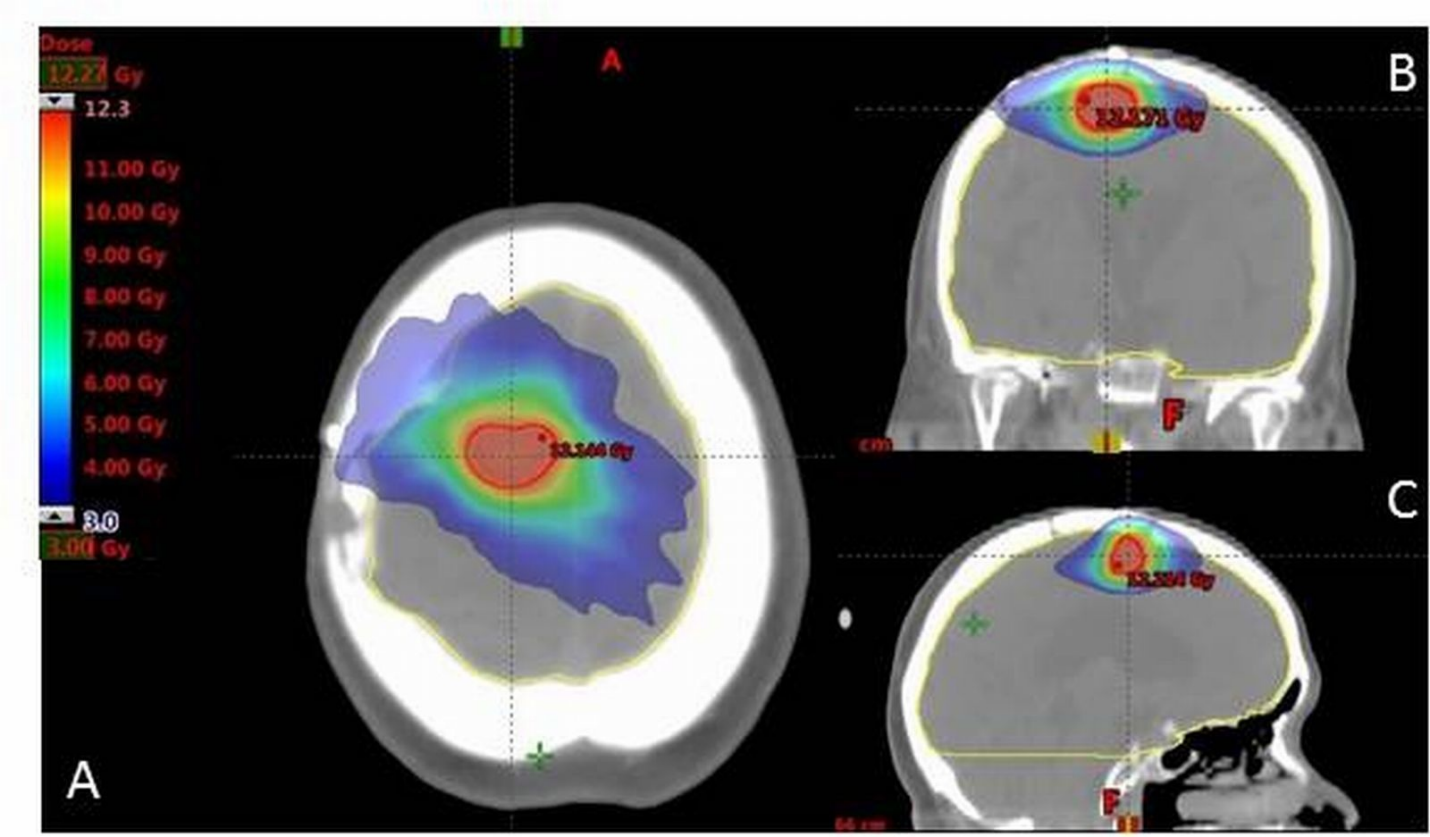
Reirradiation dose distribution. The relative radiation dose levels are indicated by colourwash (range: 3-12 Gy). A: axial section, B: sagittal section, C: coronal section

The patient well tolerated SRS and had no acute toxicity from the treatment. Approximately three months later, the first re-evaluation MRI showed stability of the two treated lesions, with no evidence of new intracranial metastases. The following second brain MRI, approximately six months after SRS, revealed a new 1 cm metastasis in the left parietal lobe. Her extra-cranial disease after 16 months from diagnosis without further systemic therapy remained as stable as neurological symptoms. In May 2012 our decision was to perform an involved field irradiation as MRI showed an initial meningeal involvement. The prescription dose was 32.5 Gy in 12 fractions (five fractions per week). She tolerated the procedure well and had no acute toxicity from treatment, even the clinical examination did not show signs of neurological suffering. Follow-up with MRI after SRS for brain metastases frequently displays local changes in the irradiated area which are often difficult to interpret, so in August 2013 on the frontal lesion and later in 2014 on the occipital lesion the patient performed stereotactic biopsy to solve histological assessment of these changes with high diagnostic accuracy. The histological examination of both lesions showed radiation-induced changes (radiation-induced necrosis) and no local tumor recurrence. The patient continued the follow-up at our department by performing brain CT scan and MRI and oncological visit every four months. In June 2016 the CT scan revealed a recurrent disease in the same area previously treated (right frontal lesion), confirmed by proton magnetic resonance spectroscopy and C-methyl-L-methionine (MET) positron emission tomography (PET). Fifty-seven months after the last irradiation and 69 months from diagnosis, a second SRS treatment, as an alternative to surgery, was proposed. The patient accepted re-irradiation and in July 2016 she underwent a second session of radiosurgery on right frontal lesion, which was delivered in the same manner as for the initial lesion, but with a single fraction dose of 12 Gy. The prescription dose was selected according to a previous study using the linear quadratic model to derive information on the cumulative biological effective tolerance dose (BEDcumulative) that results from BEDinitial plus BEDre-irradiation (Figure 2A-2C).

A stable lung disease, associated with no symptoms or signs associated with neurological decline, was documented in the following controls until October 2017 when an MRI scan showed complete remission of all metastases (right frontal, left occipital and left parietal) after 74 months by the diagnosis without further therapies.

## Discussion

NSCLC is a main cause of death from cancer and the most common source of brain metastases; prognosis of patients with brain metastases from NSCLC, despite the various treatments, remains poor with reported rate of median survival approximately of three-six months [2]. The patient presented in this case report was alive more than six years from diagnosis; data on long-term survivors are reported in the literature, especially if an oligometastatic situation is identifiable. In these patients an aggressive local treatment should be considered, evaluating carefully the probability of tumor control and the risk to develop late toxicity. Historically, whole brain radiation therapy (WBRT) has been the mainstay of treatment for many years, but the deterioration of neurocognitive function for long-term survivors who underwent WBRT cannot be neglected [3]. Stereotactic radiosurgery is a noninvasive technique delivering a single large fraction of ionizing radiation to a well defined small intracranial target with a very sharp peripheral dose falloff. SRS can have a big impact on control of brain metastases and patient survival while providing optimal sparing of healthy brain tissue and a low risk of neurocognitive deterioration. HT-based SRS is a pure coplanar radiosurgery technique which, however, provides good dose falloff and brain sparing [4]. Distinct advantages of HTSRS include built-in megavoltage CT for accurate patient positioning and powerful beam intensity modulation, a feature which can be important to improve dose conformity for complex-shaped lesions and for irradiation of previously treated sites. The issue on neurocognitive deterioration was widely debated in literature; to our knowledge, at least four randomized trials evaluated the adding of WBRT to SRS in oligometastatic brain patients; in the combined arm there was a reduced risk of local failure but no benefit in terms of survival and a decline in neurocognitive function [5-8]. It seems that the long-term adverse effects of WBRT on neurocognitive function might outweigh the benefit provided by improved local control. On this basis our patient was treated first with SRS on two lesions, considering that this was the only site of disease at that time and the patient was 52 years-old. Unfortunately an early progression was detected and a tailored conformal radiotherapy was delivered on the new lesion due to the extension of meningeal infiltration. Of note in our experience, a strictly radiological follow-up was considered advisable, and during time several changes in treated lesions were detected. This might be an issue related to the delivery of radiotherapy technique (SRS) that could make it difficult to differentiate a progression of disease from radiation-induced toxicity as radionecrosis, in fact, our patient underwent to biopsy of a treated area twice due to radiological progression which if confirmed would change the therapeutic approach. An advantage of SRS is the possibility to repeat it as salvage treatment, not combined with upfront WBRT, always in the perspective to limit the risk of neurological toxicity. Regarding neurological complication of repeated SRS, some literature data reported that repeated SRS could be performed with minimal central nervous system (CNS) toxicity [9], even RTOG 90-05 trial revealed the feasibility of SRS as retreatment of recurrent primary and metastatic brain tumors previously irradiated, with a maximum tolerated SRS dose ranged from 15 to 24 Gy depending on the tumor size [10]. Our patient underwent to a repeated SRS on right frontal lesion with a dose of 12 Gy and, in addition to an excellent response, she did not reveal any neurocognitive impairment.

## Conclusions

We presented the case of a very long surviving patient with oligometastatic NSCLC who was treated with repeated radiosurgery without whole brain radiotherapy. No late toxicity in terms of neurocognitive impairment was registered. SRS might be considered as an excellent non-invasive therapeutic option for patients with favorable biological tumor features.
